# Innate Immunity Cells and the Neurovascular Unit

**DOI:** 10.3390/ijms19123856

**Published:** 2018-12-03

**Authors:** Ivan Presta, Marco Flavio Michele Vismara, Fabiana Novellino, Annalidia Donato, Paolo Zaffino, Elisabetta Scali, Krizia Caterina Pirrone, Maria Francesca Spadea, Natalia Malara, Giuseppe Donato

**Affiliations:** 1 Department of Health Sciences, University “Magna Græcia” of Catanzaro, 88100 Catanzaro, Italy; presta@unicz.it (I.P.); elisabettascali@libero.it (E.S.); kriziapirrone@gmail.com (K.C.P.); 2 Department of Cell Biotechnologies and Hematology, University “La Sapienza” of Rome, 00185 Rome, Italy; marco.vismara@uniroma1.it; 3 Institute of Molecular Bioimaging and Physiology, National Research Council, 88100 Catanzaro, Italy; fabiana.novellino@cnr.it; 4 Department of Medical and Surgical Sciences, University “Magna Graecia” of Catanzaro, 88100 Catanzaro, Italy; annalidia.donato@gmail.com; 5 Department of Clinical and Experimental Medicine, University “Magna Graecia” of Catanzaro, 88100 Catanzaro, Italy; p.zaffino@unicz.it (P.Z.); mfspadea@unicz.it (M.F.S.); nataliamalara@unicz.it (N.M.)

**Keywords:** blood-brain barrier, innate immunity, macrophage polarization, inflammation, brain tumors, neurodegeneration, Alzheimer disease, Parkinson’s disease, multiple sclerosis

## Abstract

Recent studies have clarified many still unknown aspects related to innate immunity and the blood-brain barrier relationship. They have also confirmed the close links between effector immune system cells, such as granulocytes, macrophages, microglia, natural killer cells and mast cells, and barrier functionality. The latter, in turn, is able to influence not only the entry of the cells of the immune system into the nervous tissue, but also their own activation. Interestingly, these two components and their interactions play a role of great importance not only in infectious diseases, but in almost all the pathologies of the central nervous system. In this paper, we review the main aspects in the field of vascular diseases (cerebral ischemia), of primitive and secondary neoplasms of Central Nervous System CNS, of CNS infectious diseases, of most common neurodegenerative diseases, in epilepsy and in demyelinating diseases (multiple sclerosis). Neuroinflammation phenomena are constantly present in all diseases; in every different pathological state, a variety of innate immunity cells responds to specific stimuli, differentiating their action, which can influence the blood-brain barrier permeability. This, in turn, undergoes anatomical and functional modifications, allowing the stabilization or the progression of the pathological processes.

## 1. Introduction

The blood-brain barrier (BBB) is involved in almost all major diseases of the central nervous system. Its alterations can compromise the fundamental processes which govern brain homeostasis, such as the balance between interstitial fluid and the bloodstream, the passing across of normal and pathological cellular elements through cerebral vessels, and the immune processes that take place in the nervous tissue. In recent years, many studies have shown the relevance of the immune system, most importantly of innate immunity, with regard to BBB integrity in neurological diseases. In this paper, we will examine the role played by innate immunity cells with regard to structural and functional changes of BBB in the main categories of neurological disease.

## 2. Basic Aspects of the Blood-Brain Barrier, Cells of Innate Immunity and Cerebral Edema

### 2.1. Blood-Brain Barrier

**Figure 1:**
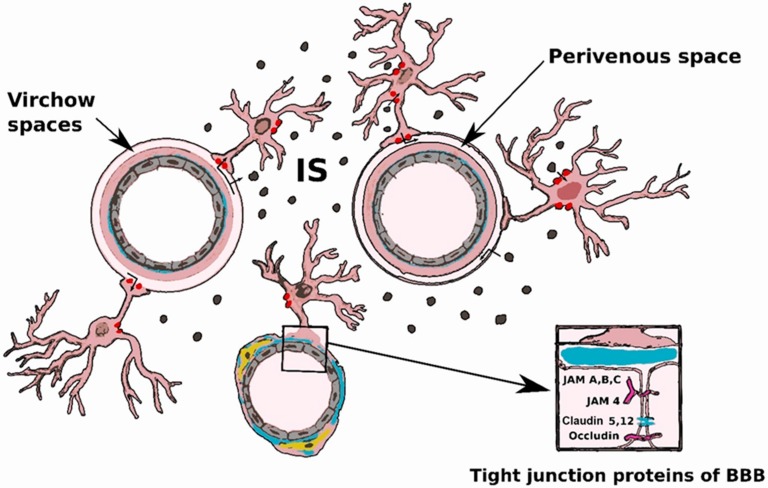
Glymphatic system and blood-brain barrier (BBB). Cerebrospinal fluid (CSF) flows into the brain from subarachnoid and cisternal spaces through periarterial spaces (Virchow spaces). CSF exchanges with interstitial fluid are facilitated by aquaporin-4 (Red dots) located on the end-foot processes of perivascular astrocyte. The CSF motion into the brain pushes the convective streaming of interstitial fluid and interstitial solute (IS) (Black dots) through the extracellular space to perivenous spaces. Perivenous fluid and solutes then drain from the brain mainly along the large-caliber ventral veins. Inset: major proteic components of tight junctions.

The blood-brain barrier anatomically and functionally separates the nerve tissue of the central nervous system from the blood. It is present throughout all of the central nervous system (CNS), including the spinal cord, except in the circumventricular organs [1]. The BBB protects the CNS, allowing the homeostasis of the microenvironment in the CNS, regulating fluctuations in electrolytes, and the passage of hormones and metabolites. The BBB, under physiological conditions, can selectively regulate the transport of large water-soluble molecules, through the capillary wall, whereas small, hydrophobic substances may pass through the endothelium by passive diffusion [2]. The BBB was classically considered the basis of the immune privilege of the brain. The BBB would inhibit the efferent arm of the immune system by hampering immune cell entry into the CNS, whereas the absence of lymphatic vessels into the brain would block the afferent arm of the immune system, including the drainage of CNS antigens into peripheral lymphatic tissues. Therefore, the local antigens were thought to be sequestered in the nervous tissue and invisible to the immune system [3]. The rediscovery of the structure and the description of the functioning of the so-called “glymphatic system” and meningeal lymphatics of the dura mater has shed new light on the concept of immune brain privilege. The glymphatic system allows cerebrospinal fluid (CSF) to flow into the brain, within periarterial spaces, and allows interstitial fluid and solute to be cleared via perivenous spaces. The transport of CSF into the brain parenchyma is facilitated by aquaporin-4 (AQP4) water channels expressed in astrocytic end-feet that unsheathe the brain vasculature (Figure 1). The interstitial fluid of the brain is collected in the perivenous space, and from there it drains out toward the cervical lymphatic system via the meningeal lymphatics. Following dural blood vessels and cranial nerves, interstitial fluid exits the cranium together with cranial nerves, arteries and venous sinuses to join cervical lymph nodes [4]. The glymphatic system and the BBB probably are complementary systems. Interestingly, sleep disturbance and inflammation may both lead to BBB and glymphatic dysfunction. BBB changes can also produce glymphatic dysfunction due to their effects on the AQP4 channels-expressing astrocytes. Indeed, harmful substances for brain can freely pass through damaged barrier and then trigger excitotoxicity and neuronal death [5]. Tight junctions (TJs) arranged between endothelial cells (ECs) are a major component of the BBB. TJs occlude cell junctions and are composed by transmembrane proteins such as claudins (Cldns) and occludins (Oclns), which are connected intracellularly to the actin filaments. TJs act to seal off the intercellular space and form a barrier between the nervous tissue and the arterial bloodstream, mainly ruled by the end feet of astrocytes, which are in contact with the basement membrane of capillaries [1]. Development of the BBB begins with embryonal angiogenesis. Vascular endothelial growth factor (VEGF) receptors (VEGFRs) and their ligand play a pivotal role in such processes. The knock-out phenotype of each of these genes leads to failure in the development of blood vessels, and homozygous mice for VEGF mutation showed a severe disturbance of this process. A dosage-dependent effect of VEGF on vasculogenesis is evident [6]. Many proteins have been identified as components of the tight junction, and knowledge of their architectural organization and interactions is critical for understanding BBB biology. Trans-membrane proteins (TMPs) interact with adaptor proteins of the cytoplasmic plaque by their C-terminal domains. Based on the number of transmembrane domains, TMPs are classified into five groups, known as tetraspan Marvel-domain proteins (occludin, tricellulin, and Mar velD3), the claudin family of proteins, the trispan BVES (blood vessel epicardial substance) protein, the single-span JAMs (junctional adhesion molecule-A, -B, and -C), and the polarity determinant Crumbs3. The cytoplasmic plaque to which transmembrane proteins bind is composed of *zona occludens* proteins (ZO-1, ZO-2, and ZO-3), MUPP1, cingulin, PATJ, PALS1, PAR3, and PAR6, which in turn connect with the cytoskeleton (Figure 1). Moreover, several signaling molecules, such as protein kinases and phosphatases, GTP-binding proteins, transcription factors, and factors acting post-transcriptionally, are associated with the proteins that compose cytoplasmic plaque [7]. In EC plasma membrane, small, bulb-shaped invaginations, called *Caveolae*, are present, containing small proteins, called caveolins, that represent a family of integral membrane proteins playing a pivotal role in endocytosis and transcytosis, and in maintaining lipid composition of the membrane. Caveolins (Cavs) may act both as positive and negative regulators of intracellular signaling with their scaffolding properties. These proteins are thought to play a role in the regulation of BBB function as they regulate the intracellular distribution of the signaling molecules. Cav-1 overexpression protects the integrity of the BBB mainly by preventing the degradation of TJ proteins in rats [8]. Pericytes are contractile cells that are enclosed within the basal lamina of the endothelium along the capillaries and post-capillary venules. They may be considered mesenchymal multipotent stem cells, which can give rise to smooth muscle cells or ECs. Pericytes play a major role in the angiogenesis and in the BBB integrity. The recruitment of pericytes and smooth muscle cells (SMCs) with extracellular matrix (ECM) production are fundamental steps in the formation of blood vessels. Pericytes show specialized characteristics and roles in different organs such as the brain, kidney and liver. Furthermore, the density of pericytes and vessel coverage is not the same in all tissues [9]. Direct contact between pericytes and ECs is present at points where the basement membrane is absent through the so-called “peg-and socket” junctions that are formed by connexin-43 hemichannels and N-cadherin. Interaction of ECs with pericytes and SMCs is of paramount importance in the regularization, stabilization and function of the BBB [9,10,11]. Astrocytes surrounding microvessels and capillaries by the end-feet of their processes interact with endothelial cells. Astrocytes are essential in the regulation of cerebral blood flow, as they transmit signals and maintain BBB function. Indeed, such types of glia can induce barrier properties and influence the polarization of transporters [9]. Astrocytes maintain BBB homeostasis by secreting diffusible factors such as retinoic acid (RA), Sonic hedgehog (Shh), angiopoietin1 (Ang-1) and glial-derived neurotrophic factor (GDNF), which interact with receptors located on ECs, in order to reduce permeability increasing junctional protein expression, raising trans-endothelial electrical resistance (TEER). Furthermore, a down-regulation of junctional proteins and a leaky BBB leads to a strong reduction of two astrocytic proteins, laminin a2 and α-dystrobrevin (α-DB), the latter being expressed in astrocytic end-feet [12]. It seems reasonable to assume that, in some way, cerebral activity may regulate the BBB functionality, although the fine mechanisms by which this happens are not completely understood; this relationship has brought about the concept of the neuro-vascular unit (NVU), which properly corresponds to the interaction between the BBB and the neuronal population. Here, astrocytes play a predominant role, with their glutamatergic synaptic activity and metabotropic glutamate receptors that route a calcium-dependent signaling cascade [13]. The non-cellular component of the BBB is mainly represented by the basement membrane (BM), a barrier working on the basis of charge and molecular weight of molecules. Astrocytes, pericytes and ECs synthetize its molecular components, such as type IV collagen, laminin, fibronectin, osteonectin, heparan sulfate and nidogen. In the brain, under pathological conditions, it has been shown that NVU cellular components such as astrocytes, pericytes and microglia, by releasing chemokines and cytokines, can promote leukocytes adhesion to the endothelial cells of the BBB and their entry into the central nervous system. In this way, in a strict sense, a cross-talk between BBB and immune system cells is realized [14].

### 2.2. Innate Immunity Cells

In this article, we focus on the role of some of the main types of innate immunity cells in neurological diseases. Notably, we have considered macrophages, mast cells (MCs), Natural Killer cells (NKCs) and polymorphonuclear neutrophils (PMNs). Innate immunity cells are activated via binding to damage-associated molecular patterns (DAMPs) or pathogen-associated molecular patterns (PAMPs) by their pattern-recognition receptors (PRRs). Also, microglia are commonly considered part of the cellular system of innate immunity, since they share with circulating phagocytes, cytokine production and ability to recognize PAMPs and DAMPs. Innate immunity comprises inducible innate mechanisms, such as cellular activation, and non-inducible innate mechanisms, mainly represented by body barriers such as epidermis, mucous membranes and the BBB. 

In other words, the so-called “innate response” is a non-specific or broadly specific mechanism aimed at avoiding entry or promoting clearance of foreign entities. Physical and chemical barriers may exclude antigens in a non-specific way, whereas innate immunity cells recognize molecular patterns common to a wide variety of pathogens or produced by host cells under stress. In this way barriers and immune cells realize a synergistic action. In another paper about this topic, we described the general characteristics of macrophages and microglia, along with the concept of macrophage polarization [15]. In brief, the tissue environment, both in physiological or pathological states, may move the macrophage cell population toward a proinflammatory M1 phenotype or toward an anti-inflammatory M2 state; this is easily evaluable by immunohistochemical techniques, highlighting the elevated expression of M1 markers like inducible nitric oxide synthase (iNOS) and interleukin-6 or, alternatively, M2 markers like arginase 1, CD206 and CD163 [16,17,18]. MCs are long-lived cells, associated with chronic inflammation and allergic reactions, that contain basic-staining cytoplasmic large granules, filled with pro-inflammatory substances. Tissue invasion or injury may cause degranulation of MCs with the release of cytokines and other molecules able to trigger the inflammatory response. MCs are classified into distinct subtypes based on their tissue of residence (mucosal, serosal, or brain subtype). In the nervous system, MCs reside on the brain side of the BBB and in the leptomeninges [19]. NKCs are lymphoid elements with cytoplasmic granules containing granzymes and perforin. They are considered part of the innate response because they are rapidly reactive and equipped with a broad spectrum of recognized antigens. Resting NKCs are mainly present in peripheral blood, spleen, liver and uterus; however, they can be quickly recruited to almost all tissues of the body. Upon activation, NKCs act against their target by producing perforins, pore-forming proteins and granzymes, which are a family of serine proteases. The synergic action of these two classes of molecules causes apoptotic and non-apoptotic death of targeted cells [20]. PMNs are the most common type of leukocytes, and can behave as both phagocytes or granulocytes. In response to injury or pathogen attacks, they massively enter tissues in response to chemokine gradients. At the inflammatory site, the PMNs are activated via their PRRs [21].

### 2.3. Brain Edema 

Brain edema (BE) consists of an increase in brain volume resulting from a localized or diffuse abnormal accumulation of fluid within the brain parenchyma [22]. This problem is a major cause of mortality in many types of brain pathologies, including trauma, cerebral infarcts, hemorrhages, infections and tumors. BE is classified into vasogenic and cytotoxic or cellular edema. Vasogenic edema (VE) is associated with functional or anatomical impairment of BBB with increased passage of plasma proteins and water into the extracellular compartment, whereas cytotoxic edema (CE) results from increased water uptake by injured brain cells. Despite this classification, in most cases there is a combination of both different types of edema, depending on the disease time course. For example, during cerebral ischemia, decreased blood flow produces a breakdown of the ionic pumps, resulting in CE; subsequently, the damage of capillary endothelium disrupts BBB and vasogenic edema occurs. CE is early observed within a few hours, and then decreases within 1 day, whereas VE starts within two to three days and is maintained for several days [23]. In meningitis and traumatic brain injury, vasogenic and cytotoxic edema coexist [22]. In brain tumors, VE is the classically associated type of edema.

Many soluble factors and functional molecules can induce BBB disruption and VE. Very often, cells of innate immunity are directly or indirectly capable of introducing these molecules into the pathologic environment.

## 3. Blood-Brain Barrier (BBB) and Innate Immunity in Brain Pathology

### 3.1. Infectious Diseases

In this paragraph, we will summarize the general mechanisms involving interactions of viral and bacterial agents with brain through the BBB and innate immunity cells. Pathogens can trigger cross-talk between cells of innate immunity and the blood-brain barrier, whereby the permeability of the barrier increases under the action of the cytokines of the cells of the immune system, and the latter can enter the nervous tissue more easily and exercise their functions. In recent years, it has been shown that most of these mechanisms are also active during non-infectious diseases of the CNS.

**Figure 2:**
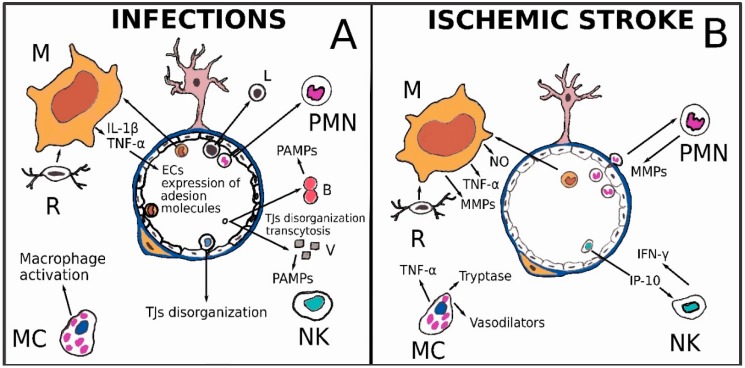
(**A**) Infectious agents can destroy or displace TJs’ constituent proteins or cross the BBB, also through the initiation of caveolin-dependent endocytosis mechanisms. Once pathogens have entered the nervous system, they are able to stimulate innate immunity cells through their PRRs. Cytokines such as tumor necrosis factor-α and interleukin-1β are secreted in the inflammatory environment and stimulate ECs belonging to the BBB to upregulate cell adhesion molecules like VCAM-1, ICAM-1 and E-selectin, allowing new immune cells to enter the nervous tissue. Other immune cells, such as lymphocytes and NK cells, can disarrange the TJs’ structure and increase vessel permeability. Mast cells releasing mediators such as histamine, tryptase or CCL5, represents another possible way of macrophage activation; (**B**) Microglia (R) is rapidly activated after the ischemic event and releases mediators such as TNF-α, superoxide anion and NO, able to recruit circulating monocytes. Microglia and macrophages (M) show a pro-inflammatory activation pattern and produce molecules involved in oxidative damage and metalloproteinases (MMPs) that cause BBB leakage. After prolonged ischemia, Polymorphonuclear Neutrophils (PMNs) can be found in infarcted parenchyma. PMNs share with macrophages and microglia the ability to produce various molecules, such as MMPs, detrimental for BBB. Mast cells (MCs) degranulate after stroke and secrete compounds capable of increasing vascular permeability. NK cells are active during the acute phase of ischemic stroke. NK cells, in cerebral ischemia, promote neural cells necrosis and damage of BBB via IFN-γ. Moreover, cytokines such as IP-10 secreted by CXCR3-activated NK cells, (a chemokine of the CXC subfamily) intensify injury to the BBB. Indeed, IP-10 expression (detectable in ischemic brain tissue) may induce NK cell infiltration following cerebral ischemia.

Bacteria can invade the central nervous system by contiguity or hematogenously. According to their characteristics of pathogenicity and virulence, bacterial agents can cause purulent lesions through (i) the recruitment and lysis of PMNs; (ii) inflammatory reactions in which mononuclear leukocytes predominate; (iii) inflammatory edema due to toxins and other substances released by the bacteria themselves; and finally (iv) lysis in the absence of bacterial replication with release of endotoxin polysaccharide of gram-negative or gram-positive glycolipopeptides [24]. The BBB is the primary defense against brain infections; in physiological conditions, it limits, in some way, the trafficking of innate immunity cells between the brain and the rest of the organism, and vice versa. However, during pathological processes, the BBB no longer seems to limit the action of elements such as neutrophils, macrophages or NKs which, during viral infections, act limiting the viral population also thanks to the production of protease, nuclease and interferon. This response is non-specific, but rapid, and can be fully active within a few hours [25]. Pathogens can access nerve tissue via a trans-cellular or a para-cellular pathway [26] (Figure 2A). Infectious agents can destroy or displace TJ-forming proteins. The viral glycoprotein gp120 of human immunodeficiency virus (HIV) can activate the C–C chemokine receptor type 5 (CCR5) and C–X–C chemokine receptor type 4 (CXCR4), expressed by ECs, causing the degradation of TJ components through the ubiquitin-proteasome system [27]. In turn, the meningococcal type IV pili bacteria can infiltrate the CNS by recruiting the polarity complex Par3/Par6/protein kinasae C zeta (PKCζ) which promote TJ formation at the luminal side of the ECs. Such an event causes the formation of ectopic intercellular junctional complexes at the site of bacteria-cerebral endothelial cell interaction, disorganizing the intercellular TJs of the lateral surface [28]. The ECs belonging to the BBB, as well as circumvented ones, can also be crossed by transcytosis-like mechanisms [26,29]. The infectious agents can also cross the BBB through the initiation of caveolin-dependent endocytosis mechanisms, exploiting EC expressed receptors such as CD155 or Necl-5 [26,30]. The West Nile virus (WNV) could cause encephalitis, entering the CNS. This virus is able to produce a loss of TJ components and elicit production of MMPs, mainly released from activated astrocytes. Interestingly, enhanced infiltration of activated immune cells into the brain, some of which may be infected, through the disrupted BBB, may facilitate a second wave of virus neuroinvasion via the so-called “Trojan-horse” route [31]. Once pathogens have entered the nervous system, they are able to stimulate PRRs of innate immunity cells, initially microglia, through their PAMPs. Cytokines such as tumor necrosis factor-α and interleukin-1β are secreted in the inflammatory microenvironment (Figure 2A) and stimulate ECs of BBB to upregulate the expression of cell adhesion molecules like Vascular Cell Adhesion Molecule 1 (VCAM-1), Intercellular Adhesion Molecule 1 (ICAM-1) and E-selectin. The contact between blood-circulating leukocytes and brain endothelial cells is then mediated via interactions between ECs cell adhesion molecules and leukocyte integrins. Subsequent passage of leukocytes in nervous tissue occurs mainly by a para-cellular mechanism by reorganization of the actin cytoskeleton and remodeling of TJs. 3D confocal imaging of monocytes migration demonstrated an early discontinuous Cldn-5 staining in endothelial TJs with a quick reseal of the gaps following leukocyte migration [32]. Microglia play a central role in infectious processes of the brain. The Toll-like receptor (TLR) family recognizes PAMPs and regulates innate immune response by microglial cells activated by microorganisms, such as viruses and bacteria. Lipopolysaccharide (LPS) starts off transcriptional activation of inflammatory genes of microglial cells. Some genes, such as Tumor Necrosis Factor alpha (TNF-α), Interleukin 1 beta (IL-1β), NF-kappa-B inhibitor alpha (IκBα) and CD14, are rapidly induced, whereas genes like those of the complement family need hours to days to be expressed [33]. The innate immune response in CNS infections develops through a network of stimuli and reactions between nerve cells, microorganisms and the BBB. In general, the answer can be seen according to two different models [26] (Figure 2A): in the inside-out model, the stimulation of PRRs by toxic molecules or by pathogens causes the production of cytokines and growth factors such as TNF-α and IL-1β which, in turn, can activate cytokine receptors expressed on endothelial cells of the BBB. The activation of these signals rearranges the gene expression profile of ECs, triggering TJ disruption and BBB opening. Furthermore, TNF-α and IL-1β activate cytokine receptors expressed by astrocytes and pericytes, which are cells that constitute the BBB. These two cell types are characterized by production of the chemokine (C–C motif) ligand 2 (CCL2), alias monocyte chemoattractant protein 1 (MCP1) and enhanced phagocytic activity, respectively. CCL2 can be transported to the luminal side of the BBB vessels by ATP Binding Cassette Subfamily B Member 1 (ABCB1) expressed on ECs, increasing leukocyte and monocyte infiltration in nervous tissue. In the outside-in model, both pathogen molecules and proinflammatory cytokines bind to their receptors, e.g., PRR, TNF receptor and interleukin 1 receptor (IL-1R), on BBB endothelial cells, and through these receptors, route their signals along the Mitogen-Activated Protein Kinase 1 (MAPK) and Nuclear factor NF-kappa-B (NF-kB) pathways, inducing TJ disorganization and the upregulation of adhesion molecules [26]. The expression of these molecules increases adhesion of leukocytes to the luminal side of the brain vessels of the BBB. The crosslink between lymphocyte function-associated antigen 1 (LFA-1), an adhesion molecule found on lymphocytes, and ICAM-1 causes reorganization of endothelial cell cytoskeleton and TJ loosening, forming a discontinuity of the BBB, and this allows leukocytes and pathogens to cross it. Finally, infiltrating leukocytes and pathogens induce the activation of other PRRs on microglia, pericytes, and astrocytes, leading to the production of cytokines and growth factors, such as TNF-α and IL-1β, CCL2, and Granulocyte-macrophage colony-stimulating factor (GM-CSF). 

### 3.2. Ischemic Stroke

Following cerebral ischemia, cytotoxic edema starts with water accumulation in astrocytes and neurons. Such events are caused by increased permeability of the cell membrane for potassium and sodium and failure of the Na^+^/K^+^ ATPase pump, accompanied by entry of osmotically active solutes. Release of glutamate from damaged neurons triggers excitotoxic mechanisms that in turn increase the intake of water, Na^+^, Ca^2+^, Cl^−^ both in astrocytes and neurons. In cellular edema, swelling of neurons and astrocytes with their processes may be observed, whereas dilated interstitial spaces are the hallmark of vasogenic edema. As we have observed above, AQP4 is particularly expressed at the interface between extracellular spaces and brain neuropil and is an important tool also for the balancing between glymphatic system and BBB. AQP4-null mice studies suggest that AQP4 plays a double and contradictory role in post-ischemic brain edema indulging cytotoxic edema and eliminating vasogenic edema-derived accumulation of cerebral fluid into parenchyma [34,35]. Moreover, in experimental studies on rodents, it was pointed out that AQP4 mRNA is decreased in the core of the ischemic brain tissue, whereas it is maximally increased in the surrounding area at three days after the ischemic stroke [36,37]. Hematogenous macrophages infiltrate into the inner border zone of the infarcts; osteopontin produced by hematogenous macrophages induces an astrocyte process extension toward the infarct border zone, which may contribute to the repair of the ischemic neurovascular unit with probable reintegration of AQP4 physiological action [38]. Circulating and resident macrophage populations play an integrated and complementary role in cerebral ischemia. Microglia are rapidly activated, after an ischemic event, by many different mediators and stimuli occurring in hypoperfused brain areas. Among the first stimuli sensed by microglia there are hypoxia, increased extracellular adenosine triphosphate (ATP) and reactive oxygen species (ROS) like superoxide anion (O_2_^−^) and nitric oxide (NO) (Figure 2B) [26,39,40,41]. In turn, activated microglial cells release mediators like MCP-1/CCL2, TNF-α, IL-1β and IL-18, which are able to recruit circulating monocytes, always by increasing the expression of adhesion molecules on endothelial cells luminal membrane. Again, activating inflammatory pathways, secreted cytokines damage the BBB and move macrophages towards a pro-inflammatory or anti-inflammatory phenotype, even if a real polarization, corresponding to a state M1 or M2, is not acquired [42]. Paradoxically, the inhibition of microglia in various experimental models of ischemic stroke leads to contradictory results [43]. The number of circulating monocytes rises early after cerebral ischemia, but the peak in the ischemic tissue is reached between three and seven days [44]. Interestingly, in male C57Bl/6J mice, pro-inflammatory Ly6C^high^ monocytes infiltrating ischemic lesion can differentiate into M2 anti-inflammatory monocyte-derived macrophages, inducing the same state to adjacent monocyte-derived macrophages or microglia and exerting an acute protective effect after ischemic stroke [45]. Microglia and macrophages are the most prominent inflammatory cells in cerebral infarction in humans. Autopsy studies have shown that microglia are lost in the necrotic core of early lesions, whereas they are increased in the surrounding penumbra zone. Macrophages in lesions are, in part, derived from the resident microglia, depending on the lesion stage. Microglia and macrophages reveal mainly a pro-inflammatory activation pattern and can produce molecules involved in oxidative damage and metalloproteinases (MMPs), detrimental for BBB structure and function (Figure 2B). At later lesion stages, the majority of macrophages show intermediate activation patterns with production of anti-inflammatory and pro-angiogenic mediators. Such findings are chronologically associated with the beginning of edema reabsorption. In normal white matter of stroke patients, as well as in controls, microglia are partly activated toward a pro-inflammatory phenotype which may be able to influence the permeability of the barrier [46]. In such a way, the peripherical component of edema can be generated.

PMNs share with macrophages and microglia the capability of producing many of such molecules (Figure 2B). Rolling and adhesion have been described early after stroke in brain vessels [47], following the up-regulation of chemo-attractants and adhesion molecules [48]. As happens in other sites of the organism, neutrophils are the first population recruited at the site of tissue damage, followed by the recruitment of monocytes. The initial recruitment of neutrophils is due to the production of chemokines such as C-X-C Motif Chemokine Ligand 8 (CXCL8) by resident macrophages [49]. In humans and in experimental models, it has been demonstrated that during the acute phase of reperfusion, neutrophils remain within the boundary of the neurovascular unit and leptomeningeal spaces, while they are not found in the brain parenchyma; only after prolonged ischemia can PMNs be found in infarcted parenchyma [43,50]. Such a finding suggests that PMNs may play a precocious role in the leakage of the BBB. Both PMNs and macrophages produce MMPs, and because of their capability to degrade the ECM components and tight junctions, they have been implicated in post-stroke BBB leakage (Figure 2B). Opening the BBB follows a biphasic trend: the more precocious alterations occur within hours of stroke onset, the later ones are present after 24–48 h. Such early and late alterations in barrier permeability are consistent with the increased expression of MMP-2 and MMP-9, which are the main MMPs that have been shown to be active following stroke. In particular, the first type of alteration is reversible and is attributable to MMP2 activity on endothelial cells; the second type of alteration is attributable to MMP9 and is associated with a complete degradation of the basal lamina [51]. Interestingly, MMP inhibitors such as BB-1101 and MMI270 reduced BBB hyperpermeability and brain edema in experimental animals after cerebral ischemia [28]. It has been seen that MCs play a role in ischemic brain stroke. Such types of cell are resident in brain tissue and meninges and can react very promptly, even before microglia [52]. MCs degranulate after stroke and may act on basal membranes, inducing BBB leakage, extravasation of fluid, and promoting brain edema. MCs may secrete many classes of molecules able to increase vascular permeability such as vasodilators (heparin, histamine, serotonin, nitric oxide, vasoactive intestinal peptide and calcitonin gene-related peptide), proteases (tryptase) and cytokine (tumor necrosis factor alpha, TNF-α) [53]. Since TNF-α exposure induces a prolonged expression of intercellular adhesion molecule-1 (ICAM-1) on endothelial cells, cytokine secretion by MCs can favor the further entry of other blood cells into the ischemic tissue [19]. NK cells are active in the acute phase of ischemic stroke [54]. In human ischemic brain, NK cell infiltration can be observed, and it peaks between 2 days and 5. In a mouse model of C57/BL6 wild type strain with permanent middle cerebral artery occlusion (pMCAO), the NK cells’ infiltration into the infarct region reached its highest levels within 12 h after ischemia. NK cells, in cerebral ischemia, promote neural cells necrosis and damage of BBB via the production of IFN-γ. In vitro studies confirm that NK cells may have a negative impact to the permeability of the BBB. Cytokines such as IP-10 (a chemokine of the CXC subfamily secreted by CXCR3-activated NK cells,) intensify injury to the BBB. In turn, IP-10 expression (detectable in ischemic brain tissue) may induce NK cell infiltration following cerebral ischemia [55] (Figure 2B).

In conclusion, innate immunity cells, together with direct ischemic vascular damage, cause cerebral edema in conjunction with loss of BBB functions. Topographic and chronological differences in activation may occur after the ischemic event for each type of cell. 

### 3.3. Brain Tumors

**Figure 3:**
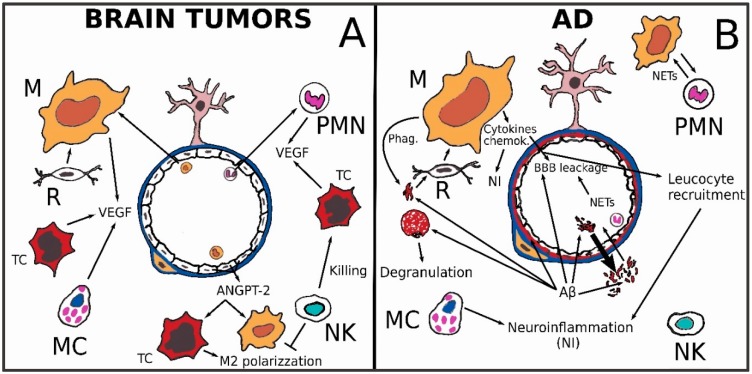
(**A**) The structure of neoformed vessels in most important brain tumors, such as glioblastoma, is often abnormal with permeability of BBB. VEGF is the major angiogenetic factor: it can be produced by cancer cells (mainly in hypoxic areas), MCs, PMNs and Macrophages. VEGF acting on TJs proteins synthesis, can enhance vessels permeability. Endothelial angiopoietin (ANGPT2) promote the recruitment of proangiogenic, M2-polarised macrophages. In endothelial cells the Ang/Tie pathway can regulate pathological vascular remodeling and vascular permeability during the processes of inflammation, tumor angiogenesis and metastasis. The same pathway can also be activated in macrophages and cancer cells. NK cells in GBM may exert a preferential killing activity of GBM stem-like cells. NK cells, by cytokine secretion, could be able to exchange TAMs polarization from anti-inflammatory to pro-inflammatory phenotype; (**B**) In subjects affected by sporadic Alzheimer’s disease (AD) Aβ amyloid accumulation is due to a reduced clearance from the brain, ascribable to reduced metabolism, diminished efflux across the BBB or decreased CSF bulk flow through glymphatic system. Both brain-derived and peripheral Aβ amyloid are transported through BBB by receptor-mediated transcytosis. Microglial cells can be activated by Aβ fibrils interacting with several receptors, thus inducing the production of pro-inflammatory mediators. Neutrophil Extracellular Traps (NETs) are fibers of decompacted chromatin decorated with antimicrobial proteins and histones originating from PMNs that can contribute to the activation of microglia and BBB leakage. Aβ amyloid causes MCs degranulation contributing also in such a way to neuroinflammation (NI).

Tumors such as glioblastomas (GBMs) are considered immunologically “cold” and their microenvironment is defined as an “immunological desert”. In this inhospitable territory, cells of innate immunity dominate. In malignant brain tumors, angiogenesis is favored by tumor cells and cells of innate immunity. The neo-generated vessels will be abnormal, exhibiting alterations of permeability capable, in turn, of encouraging the movement of monocytes or circulating PMNs in the tumor tissue. This will favor the formation of cerebral edema and sometimes, paradoxically, will prevent the passage of drugs. The endothelial cells of newly formed vessels are capable, in turn, with macrophage and neoplastic elements, of constituting a perivascular niche, functional to their needs (Figure 3A).

BBB breakdown is an important event both in primary and in metastatic brain tumors. Brain edema volume is a prognostic factor both in gliomas and in secondary tumors [56,57]. In another paper about this topic, we described a high infiltration extent of microglia/macrophages in malignant gliomas such as glioblastoma [15]. Dexamethasone (DEX) is a widely used synthetic glucocorticoid that leads to activation of glucocorticoid receptor (GR). It has been shown that DEX administration can reduce brain edema in patients with intracranial tumors [58,59]. DEX can exert its anti-edema power acting on inflammatory responses. Moreover, DEX increases angiopoietin-1 levels, which contributes to the BBB structure stabilization, and also decreases the VEGF levels in pericytes and astrocytes via GR activation [23]. It is well known that in growing tumors, oxygen and nutrients, transported through blood circulation, becomes progressively inadequate. Hypoxic regions can induce angiogenesis by activating hypoxia-inducible factors (HIFs) that in turn can upregulate the VEGF (Figure 3A). The structure of neoformed vessels in most important brain tumors is often altered together with permeability of BBB, and chronic or cycling intermittent hypoxia is also common in non-necrotic areas [23]. Neoangiogenesis can build multiple small vessels forming glomeruloid masses that represent a classic histological feature in glioblastoma and in other primary CNS neoplasms. Endothelial multilayered proliferation and thrombosis of small vessels are other characteristic features that could be recognized. It is well known that in high-grade brain tumors, the tumor-associated macrophages (TAMs) play a pivotal role in neoangiogenesis [23]. VEGF drives the proliferation and migration of endothelial cells in various tissues, including brain. In the central nervous system, VEGF secretion is observed in astrocytes, neurons and endothelial cells, but also in glioma cells and in microglia/macrophages [15,23,60]. Interestingly, VEGFs are also known to enhance BBB permeability by disrupting tight junction-regulating proteins [23,56]. Treatment with VEGF-A induces down-regulation of *CLDN5*5 and *OCLN* genes in human ECs. Similarly, a decrease in *CLDN5* and *OCLN* expression has also been observed in mice cerebrum when VEGF-A was administered [23,61]. In GL261 glioma cell line from C57BL/6 mice, it has been demonstrated that VEGF is a modulator of the innate immune response instigating suppressive effects acting on the immunologic and pro-angiogenic function of microglia/macrophages. Such a mechanism is probably part of a regulatory feedback circuit that, through high levels of VEGF, can avoid the TAM accumulation in the perivascular niche, and concomitantly may downregulate the production of their pro-angiogenetic factors [62].

Besides neoplastic cells, endothelial cells, PMNs and TAMs, MCs can also produce VEGF, together with other mediators effective in causing BBB permeability, such as histamine, IL-8 and tryptase [63] (Figure 3A). The activation of angiopoietin/Tie-1 or Tie-2 pathway in endothelial cells can regulate pathological vascular remodeling, vascular permeability during inflammation, tumor angiogenesis and metastasis. Angiopoietin/Tie-2 signaling is emerging as a new drug target in tumors including glioma, which is intended to block the vasculature development. Such angiogenic signaling pathways can interfere with endothelial barrier properties (Figure 2B). Recently, Ang-2/Tie2 signaling has been associated with cancer inflammation because it may promote the recruitment of proangiogenic, M2-polarized macrophages (Figure 3A). These findings could be also translated to glioma cases where leakage of the BBB is prominent in some areas like the core region, and the NVU remains functional in other regions, like the invasive zone [12]. The higher the tumor grade, the more MCs infiltrate the stroma and the BBB area in human gliomas. In this way, such cellular elements may cooperate in angiogenesis likewise in other types of neoplasms by means of the enzymes and angiogenic factors that they are able to pour into the external environment [47,64]. Edema represents an important prognostic factor in patients with meningiomas [65]; its emergence is proportional to the histological grade, and it seems also to be influenced by the histotype [66,67], and to depend on the secretion of VEGF [68]. Interestingly, MCs are involved in peritumoral brain edema formation in meningiomas. MCs contain a lot of granules and are able to secrete many mediators, including VEGF, and various chemokines and cytokines, some of which are known to cause leakage of BBB. MCs are present in as many as 90% of all high-grade meningiomas and are mainly found in the perivascular areas of the tumor. A correlation between MC copiousness, VEGF secretion and peritumoral edema in meningiomas is very likely [69,70]. 

NK cells are cytotoxic lymphocytes, capable of direct killing without prior immunization [71]. Experimental studies have demonstrated that TAMs can inhibit NK cell activation and concordant cytotoxicity against tumor cells in a contact-dependent manner via TGF-β [72]. 

Purified NK cells, employed in GBM treatment, may exert a preferential killing of GBM stem-like cells, which may show potential tumoral or perycytic differentiation [73,74]. Moreover, the immunomodulation capacity of NK cells through cytokine secretion could skew TAMs from anti-inflammatory to pro-inflammatory phenotypes [75]. These last properties could be essential for successful immunotherapy against GBM and for properly modulating BBB permeability to a convenient state for chemotherapeutic treatments. In this way, diverse new drugs could enrich the panel in use for therapy. For example, the proteasome inhibitor bortezomib is effective for a variety of tumors, but not for GBM, notwithstanding the overexpression of certain component of the ubiquitin-proteasome system has been demonstrated in this neoplasm [76]. Bypassing the blood-brain barrier by means of an osmotic pump, it resulted in an increased efficacy of bortezomib in an orthotopic GBM murine model [77]. Some naturally occurring substances are known for their ability to regulated BBB in tumors interacting with innate immunity cells [78]. PMNs are a target of anti-VEGFA therapy, as they are the main producers of this proangiogenic cytokine, which, let us remember, can regulate the permeability of BBB [79]. Neutrophil-to-lymphocyte ratio (NLR) is usable as a biomarker for systemic inflammatory disease. Large-scale meta-analysis revealed that NLR is associated with an adverse clinical outcome in many solid tumors including gliomas. As frequently happens in the context of patients affected by other cancer type, glioma patients usually have a strong neutrophilia and NLR higher than 4, and this value has been associated with poor prognosis when measured before treatments. Moreover, there is a correlation between NLR value, the extent of neutrophil infiltration, the glioma grade and the acquired resistance to anti-VEGF therapy. Interestingly, NLR is also associated with high frequency of brain metastases in other solid tumors [80,81].

### 3.4. Alzheimer’s Disease

When talking about neurodegenerative diseases, we are referring to: Alzheimer’s disease (AD), Parkinson’s disease (PD), Amyotrophic lateral sclerosis (ALS), and Huntington’s Korea (HK). These conditions share many cellular and molecular aspects with aging, so the use of inflamm-aging [82], as an extension of the “network theory of aging”, has begun. To dive into this concept, it can be explained as a chronic inflammatory state, partly dependent on an augmentation in damage-associated molecular patterns (DAMP) that activate pattern recognition receptors (PRRs) of innate immunity. PRRs, such as the Toll-like receptor (TLR) family and nucleotide-binding oligomerization domain-like receptors (NLRs), are expressed in all neurovascular unit cells, and not only in the innate immunity cells. A recent review [83] reported what is known about the relationship among activation of PRRs expressed by cells of the NVU/BBB, and chronic neuroinflammation. TLR2, TLR4, NLRP1 and NLRP3 are the most important DAMP-sensing PRRs in the brain, which are activated during physiological and pathological conditions in astrocytes, microglia, neurons, and possibly pericytes and endothelial cells. Because of its heavy metabolism, represented by a weight %/oxygen consumption % ratio of 2%/20%, the brain needs special logistics to deliver the right amount of O_2_, and this is represented by a very dense capillary system with an average distance of 40–50 µm between neighboring capillaries. BBB breakdown leads to entry of neurotoxic serum proteins into the brain tissue, contributing to triggering and sustaining neuroinflammation following most of the pathways described above. Concerning AD, it has been found that neuroinflammation might be considered not only a consequence, but the cause of AD inception and progression [84]. Some authors have described the brain milieu in AD and other neurodegenerative diseases as in a “moderate, but persistent, cytokine storm” [85]. A recent study [86] pointed out that mitochondrial dysfunction, inflammation, and insulin resistance are “common molecular denominators that connect type 2 diabetes (T2D) to AD”. Both obesity and T2D are known risk factors for AD, and those conditions share peripheral inflammation and systemic insulin resistance. Both features could be explained by an overproduction of pro-inflammatory mediators (e.g., TNF-α) and low-grade chronic inflammation. In an AD brain, toxic amyloid-β oligomers (oAβs) are localized within synapses and trigger pro-inflammatory signaling [87], “priming” microglial cells and promoting their activation with the induction of pro-inflammatory cytokines and chemokines [84] (Figure 3B). This causes a synaptic deterioration in the hippocampus due to a decrease in glucose use and insulin resistance, thus leading to memory impairment, as observed in both AD and T2D [88]. Microglial cells can be activated by fibrils and oAβs interacting with several receptors, such as CD14, CD36, CD47, α6β1 integrin, and TLR4, thus inducing the production of pro-inflammatory mediators [89,90,91,92]. Microglia are also involved in extracellular Aβ degradation through the release of proteases and in phagocytosis of Aβ fibrils [93,94] (Figure 3B). In the mild cognitive impairment that precedes AD, the inflammatory changes are already present [95]. The observation of an immune dysregulation involving toll-like receptors as TLR2, TLR4 and TLR7 both in aging and in AD suggests strong importance of the innate immune background for the development of AD [96]. Moreover, the TLR-mediated activation of the immune system is now considered a possible target for pharmacological treatment in neurology and psychiatrics [97]. Microglial activation, the process at the basis of AD [98], is thought to anticipate AD’s clinical onset by many years. This is probably the reason therapies aimed at decreasing inflammation in patients with mild cognitive symptoms or established dementia gave confusing or disappointing results [99,100,101,102,103]. From an immunological point of view, AD could be modeled as different phases of innate immune activation:

- early phase: excessive pro-inflammatory microglial activation that is maybe even precedent to AD’s clinical onset;
- late phase: substantial microglial impairment.

A speculative model may consider a first phase of immunological burst caused by the presence of extracellular Aβ deposits, followed by a state of “immunological paralysis” in microglia, characterized by the inhibition of pro-inflammatory responses, a lowered TLR4 expression, and impaired phagocytosis, along with an up-regulation in anti-inflammatory cytokine production. The transition between the two phases may be due to the inability of microglia to clear the harmful stimulus and consequently the persistence of the same “bad signal” in the extracellular environment that “paralyzes” the immune system. There are several pieces of evidence to support this theory; in the brain of AD patients, compared to healthy aged controls, the immune/inflammation gene expression profile is far lower [96].

BBB integrity and function are nowadays considered crucial for brain health; the dynamic blood-brain barrier has recently been reviewed [32], as well as the cross-talk between microglia and BBB [104]. Aging deteriorates the BBB; in experimental studies it has been shown that the leakiest part is usually the section shielding the hippocampus. This is also the place where AD plaques formed [105]. Experiments with animal models of AD have suggested that, when the BBB is unable to prevent the entry into the CNS of invading pathogens such as viruses, bacteria, fungi, etc., the brain’s defense is forced to react with production of Aβ, which then wraps invaders in a predominantly amyloid, sticky, dense mass. The result is the accumulation of plaques typical of AD [106]. Van de Haar and colleagues [107] studied patients with early AD associated with cognitive decline compared to age-matched control subjects using a dynamic, contrast-enhanced magnetic resonance (MR) with a dual time resolution imaging system. They found that global leakage of BBB occurred in the AD group, in contrast with controls. They concluded that “a compromised BBB may be part of a cascade of pathologic events that eventually leads to cognitive decline and dementia” (Figure 3B). BBB breakdown is more pronounced in AD patients carrying the apoE4 allele, the most prominent genetic risk factor for AD [108]. Approximately 25% of all individuals are carriers of one or two copies of the apolipoprotein E4 (apoE4) allele. In apoE4 homozygotes, the risk of developing AD is increased by about 1500%; in the heterozygotes, the increased risk is about 400%. A significant increase in amyloid plaques in the brain at earlier ages is associated with apoE4 expression, compared with apoE2 and 3. ApoE4 impairs Aβ clearance from the brain and across the BBB [95,109]. Among brain-resident innate immune cells, microglial cells sense danger stimuli, such as proteins like Aβ, and become activated by neuronal damage, like that caused by LPS. The resulting reactive microgliosis has been implicated in generating the chronic form of microglial activation believed to promote AD’s development [110] (Figure 3B). Two different genome-wide association studies (GWASs) of sporadic AD cases indicated variants in genes related to the innate immunity as causative [111,112]. Aβ overproduction was thought initially to be the main cause of Aβ accumulation. Recent papers have suggested that in sporadic AD cases (the vast majority), Aβ accumulation is due to a reduced clearance of Aβ from the brain [113], and this may be ascribable to reduced metabolism, diminished efflux across the BBB, or decreased CSF bulk flow with involvement of the glinfathic system [114,115]. Parenchymal Aβ levels are supposed to be regulated by BBB transporters. The main receptors for Aβ transport from brain to blood are permeability glycoprotein 1 (P-gp) and the low-density lipoprotein receptor-related protein 1 (LRP-1) [116].

### 3.5. Epilepsy

**Figure 4:**
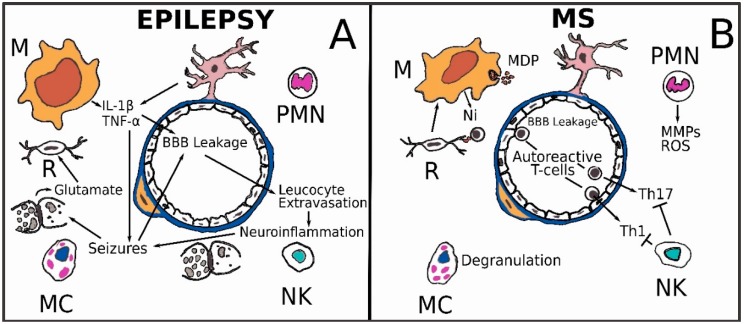
(**A**) A relationship of reciprocal induction may exist between epilepsy and neuroinflammation. Seizures cause BBB leakage that allows inflammatory cells to enter the brain tissue and release excitatory amino acids. Neuroinflammation (Ni), in turn, causes seizures altering neuronal excitability; (**B**) A precocious BBB leakage, both paracellular and transcellular, is present in multiple sclerosis (MS). Autoreactive T cells, among helper subsets T_H_1 and T_H_17, burst into the nervous tissue and may be activated by myelin debris (MDP) presented from microglial cells or inhibited by NK cells. Macrophages phagocytosis of MDP contribute to the activation state of pro-inflammatory macrophages that produce cytokines. PMNs can increase permeability of the vascular barrier by ROS and MMPs, Microglia activation is a primary feature of MS.

In recent years, the relationships between epilepsy, neuroinflammation and BBB have begun to be clarified. In particular, it has been shown that cells of innate immunity and cells belonging to the NVU can be involved in the genesis of seizures or epileptic syndromes not caused by specific infectious, autoimmune or other neuropathological alterations [117,118]. Neuroinflammation may affect seizure. Proinflammatory factors such as IL-1β, TNF-α, IL-6, Cyclooxygenase-2 (COX-2), prostaglandin E2 (PGE2), High mobility group protein B1 (HMGB1), TLR4, TGF-β and Cytochrome B-245 Beta Chain (NOX2) play important roles in seizure severity and recurrence [119,120,121,122]. It is probable that a relationship of reciprocal induction exists between epilepsy and neuroinflammation (Figure 4A). Many data were provided by brain tissue from patients with temporal lobe epilepsy (TLE) and hippocampal sclerosis (HS) and from experimental mouse models, simulating TLE. Such studies demonstrate that the increase in epileptic brain of IL-1β and TNF-α in microglia and astrocytes is followed by a cascade of downstream inflammatory steps which can lead to recruitment of cells of the adaptive immune system; on the other hand, IL-1β and TNF-α may alter neuronal excitability and the generation of seizures and may contribute to neuronal cell loss, astrogliosis and BBB damage [119]. The presence of granulocytes at an early stage and monocytes/macrophages in areas of neuronal loss both in the experimental models and in human epileptic tissue suggest that these cells may contribute to neuronal injury by releasing cytotoxic mediators, whereas NK cells or cells of adaptive immunity such as T and B were not detected in brain parenchyma in chronic rat or human epileptic tissue and during epileptogenesis in experimental models [119]. Seizures may also cause BBB leakage, which can stimulate and perpetuate neuroinflammation by extravasation of leukocytes and inflammatory molecules from blood vessels into the brain tissue (Figure 4A) [123,124,125]. In summary, initial status epilepticus can trigger acute immune and inflammatory responses within the nervous tissue, while the subsequent spontaneous recurrent seizures may cause the chronic neuroinflammation. The link between inflammation and epileptic seizures has led many authors to hypothesize a pharmacological intervention on inflammation aimed to treat epilepsy. Evidence from preclinical and clinical studies suggest that targeting inflammatory signaling pathways represents a possible complementary approach to the current symptomatic therapies in order to control seizures, particularly in forms of epilepsy that are refractory to conventional treatments [126]. This type of strategy can target a very large number of objectives linked to the cells of innate immunity. For example, the metabotropic glutamate receptors expressed by microglia cells. Metabotropic glutamate receptors (mGlu receptors) are G-protein-coupled receptors, expressed on neurons and glial cells; mGlu receptor activation may play a similar role in the nervous and the immune system, counteracting negative glutamate effects. Microglial cells express mGlu2, mGlu3, mGlu4, and mGlu5, which can differentially modulate microglial activation and function; the activation of these receptors can be triggered by the excitatory amino acids released during epileptic seizures [127,128]. 

The mammalian target of rapamycin (mTOR) signaling pathway is a regulator of cell metabolism and growth. Deregulation of the mTOR pathway has been observed in many human diseases. Rapamycin, an antifungal metabolite produced by *Streptomyces hygroscopicus* from a soil sample of Easter Island, is a specific inhibitor of mTOR, which has been shown to be useful in the treatment of various diseases [129]. Interestingly, in an experimental model in rats, rapamycin improves BBB function during the chronic epileptic phase by a reduction of local brain inflammation and blood vessel density that can contribute to a milder form of epilepsy by suppressing the inflammatory response [130]. Moreover, reduction of brain inflammation was also observed in a model of absence epilepsy [131,132].

### 3.6. Multiple Sclerosis

Multiple sclerosis (MS) is a chronic, autoimmune, inflammatory disease of the CNS that affects the brain and spinal cord. The disease results in a process of myelin sheath destruction, called demyelination, which leads to the formation of lesions named “plaques” and clinical symptoms linked to their location. The substantial heterogeneity in clinical manifestation, in disease progression, and in CNS tissue lesions makes MS a multifaceted and still-enigmatic disease. MS plaque is a peculiar, well-demarcated, multi-focal lesion of white matter, which never occurs in other inflammatory disorders of the CNS; thus, it is considered the MS hallmark [133]. Several basic processes drive the formation of plaques: inflammation, myelin breakdown, astrogliosis, oligodendrocyte injury, neurodegeneration and axonal loss, and remyelination [134]. The pathogenesis of MS plaques is highly complex (Figure 4B). Despite the efforts made in past years, much remains to be clarified on the cascade of phenomena triggering MS development. At the moment, the identification of a single cause underlying its etiology remains elusive; however, it is agreed that genetic factors and environmental influences produce a complex interaction with immunological factors, ultimately activating the autoimmune response [135]. In this context, the debate about the action of adaptive and innate immunity in MS is still open. MS has traditionally been considered an adaptive immune response through the activation of populations of T-cells (both CD4+ and CD8+ T cells) specifically identifying myelin fragments that induce tissue damage and contribute to lesion evolvement [136,137]. In the last few years, the innate immunity in MS has raised growing interest, and it is now considered to be crucial in both initiation and progression phases of MS [138,139]. Mononuclear phagocytes (i.e., microglia and macrophages) are the prevalent components of the innate immune system found in MS lesions in both relapsing–remitting and progressive phases of the disease. It has been proposed that these cells could (i) indirectly act through the activation of the myelin-reactive T lymphocytes by antigen presenting cells (APCs); (ii) directly cause neuro-inflammatory tissue damage, [140,141]; and (iii) release myeloperoxidases and reactive oxygen species that further increase the neurotoxicity [142,143]. The action of the microglia/macrophages is driven by several pro-inflammatory chemokines and cytokines [144]. Almost all classes of TLRs (TLR 1–9) are expressed on the surface of microglial cells, and the activation of these receptors is a crucial point for generation of the neuroimmune responses [145,146,147]. Several studies have demonstrated the importance of TLR expression in MS pathology, both in patients with MS and in the experimental autoimmune encephalomyelitis (EAE), the murine model of demyelinating disease) [148,149]. Indeed, the stimulation of the different TLRs with specific ligands activates macrophages and supports a sequence of effects in glial cells that results in an increased expression of the MHC classes I and II, as well as in the secretion of cytokines of the innate immune system (IL-12, IL-6, IL-1, IL-10, and TNF) and chemokine (CXCL-10, a chemoattractant for pro-inflammatory T cells), which are strongly associated with MS pathogenesis [146]. These cytokines promote several key functions in the cascade of immune response, including: (i) blood brain barrier leakage; (ii) lymphocyte attraction to sites of inflammation; (iii) activation of pro-inflammatory state; (iv) modulation of adaptive immunity; and (v) leukocyte extravasation across the blood brain barrier (Figure 4B). Other observations also lay a role for TLR response in MS pathogenesis. Immunohistochemical evaluation of brain and spinal cord samples of MS subjects revealed increased expression of TLR3 and TLR4 [149]. Moreover, in MS lesions, the oligodendrocytes showed an increase in TLR2 expression, which has been related to hindrance of tissue remyelination [150]. Finally, peripheral blood mononuclear cells (PBMCs) extracted from MS patients demonstrated a hypersensitivity to TLR4 stimulation, being capable of activating a pro-inflammatory response [151]. Pathological analysis of plaques in MS demonstrated the presence of mast cells in inflammatory infiltrates (Figure 4B). In addition, the same cells were also found in normal-appearing white matter [152,153]. Increased levels of tryptase (the specific enzyme of this cellular class) was found in the CSF of MS subjects [154]. Moreover, an increment of expression of mast-cell specific genes was found in MS plaques [155]. This evidence supports the hypothesis that mast cells play various roles in MS pathogenesis, influencing the innate immune response both in peripheral tissues and in CNS. Mast cells are activated in early disease and express mediators that affect BBB integrity and recruit T cells for pathogenic activation [156], giving life in the meningeal compartment to a complex interplay between resident mast cells and autoreactive T cells. Mast cells are elicited in the production of caspase-1-dependent IL-1β, and this mediator in turn stimulates the production of GM-CSF by T cells [157], a cytokine essential for T cell activation itself and for recruiting inflammatory monocytes into the CNS [158,159,160,161]. It has been demonstrated that the mast cells are able to generate a mature form of IL-33 [162], which is a multifunctional cytokine which belongs to the IL-1 superfamily. IL-33 is considered the most potent activator of non-cytotoxic innate lymphoid type 2 cells [163] and acts like an alarmin if released by a degraded tissue [164]. In this context, the non-cytotoxic innate lymphoid type 2 cells, stimulated by IL-33, would act not as a disease-promoter, but as a disease-silencer. A recent, interesting finding regarding the different response in terms of IL-33 production in male and female mice, expressing the disease model, laid down a convincing body of evidence supporting a differential response linked to sexual hormones. Direct evidence for testosterone actions on IL-33 induction in mast cells come from studies with male- and female-derived bone marrow mononuclear cells (BMMCs) [165]. Testosterone selectively induces IL-33 in male-derived bone marrow mast cells, and not in the female-derived ones. On the contrary, in female mice, a decreased IL-33 expression reduces the non-cytotoxic innate lymphoid type 2 cell activation and promotes the disease susceptibility [156]. This new evidence adds new insight into how some cells of innate immunity can play either a harmful or protective role, depending on the particular contexts in which they act, and how differences linked to gender could interact with immune response, leading to disease development. Mast cells are considered a potentially useful target for pharmacological strategies in the treatment of MS. Hydroxizine, an anti-histaminic drug [166], and luteolin, a blocker of activation of mast cells, were successfully employed experimentally [167,168]. The role of NK cells in MS still remains not completely clarified. Indeed, although some evidence suggested that NK cells might play a role in promoting MS and EAE, other studies demonstrate a potential protective effect of NK cells. The most numerous studies on the role of NK cells in producing MS came from EAE murine models. A body of evidence supports the detrimental action of NK cells on the CNS. In particular, a study demonstrated a worsening effect on EAE that was dependent on the presence of NK cells [169], and, consistent with this finding, another study showed that NK-depleted mice developed a milder form of EAE in comparison with those in which NK cells were expressed [170]. On the other hand, an important body of studies demonstrated a protective role of NK cells in mouse or rat EAE [171,172,173]. Studies in humans confirmed both contradictory findings. A pathological study showed that NK cells are present in demyelinating lesions of patients with MS [174]. Moreover, a reduction of cytotoxic activity of NK cells has been reported in MS subjects during the relapse phase of the disease [175,176]. However, on the other hand, other studies have suggested a beneficial effect of various subsets of NK cells in multiple sclerosis [177,178]. These discrepancies may be caused by the heterogeneity of NK cells, which include distinct NK subpopulations, each of which, performing various functions, could probably influence, in a peculiar way, the immune response. Indeed, an increase of the NK2 cell subtype was found in MS patients in the remission phase compared to patients in the relapse phase, thus suggesting that this subpopulation may promote a protective role in MS [177]. NK2 cells were also found to depress the activity of antigen-specific autoreactive T cells (Figure 4B) [178]. Taken together, these data demonstrate that NK cells may have either disease-promoting or disease-protecting effects based on the different subtypes that are activated by the microenvironment. In summary, several pieces of evidence in the literature demonstrate that the cells of innate immunity play a key role in the pathogenesis of MS and that, based on the particular microenvironment within which they work, they could act either as disease-promoting or -protecting factors, realizing a complex immune-modulating effect, still to be clarified in many aspects. 

## 4. Discussion

Taking into account what we described previously, it would appear clear that interactions between the BBB and innate immunity cells take place in all types of neurological diseases through the mediation of neuroinflammatory events, and this influences the pathogenesis and course. Although the basic mechanisms have common ground, the cells of innate immunity and the BBB/NVU are producers and targets of completely different stimuli depending on the type of disease in progress. A very interesting point is related to the fact that the innate immunity cells must often interact with each other to carry out their functions. These interactions are essential to triggering the immune response in infectious diseases at the whole body level, including the nervous system [179]. However, in this field, little is known about other neurological diseases. These interactions are probably decisive in regulating the behavior of individual cell types of innate immunity in relation to the BBB. Immunotherapy in neurological diseases can be driven by the need to enhance the immune response as in primary and secondary neoplasms or to depress it as in multiple sclerosis [180,181]. In other fields such as neurodegenerative diseases, immunotherapy is starting to become a real possibility, considering certain studies we have examined [182,183]. Interestingly, in this context, the permeability of the BBB can be a determining factor in the success of immunotherapy itself, and in turn, immunotherapy may change the status of the BBB. Indeed, the BBB is dynamically regulated by cytokines. Proinflammatory cytokines such as TNF-α, IL-1 and IL-6, released because of innate recognition of pathogens through PRRs, can weaken the BBB. On the other hand, type I and type III IFNs can be induced in response to viral infections and stabilize the BBB, preventing entry of vascular contents into the CNS. Moreover, signaling through TAM receptor tyrosine kinases (Tyro3, Axl, and Mertk) on ECs has been shown to enhance BBB integrity in mice and humans [184]. In summary, the cross-talk between the BBB and innate immunity cells, through neuroinflammation, plays a central role in all neurological diseases. Its knowledge is increasingly taking on translational value for innovative therapies that could radically change patients’ prognosis in the future.

## Abbreviations

BBB	Blood-brain barrier
BM	Basement membrane
BVES	Blood vessel epicardial substance
CNS	Central nervous system
CSF	Cerebrospinal fluid
DAMPs	Damage-associated molecular patterns
EAE	Experimental autoimmune encephalomyelitis
ECM	Extracellular matrix
GDNF	Glial-derived neurotrophic factor
JAMs	Junctional adhesion molecule
MMPs	Metalloproteinases
NVU	Neuro-vascular unit
PAMPs	Pathogen-associated molecular patterns
pMCAO	Permanent middle cerebral artery occlusion
PMNs	Polymorphonuclear Neutrophils
PRRs	Pattern-recognition receptors
SMC	Smooth muscle cell
TAMs	Tumor associated macrophages
TMP	Trans-membrane protein
